# Cingulate circuits are associated with escalation of heroin use and naloxone-induced increases in heroin self-administration

**DOI:** 10.1016/j.addicn.2021.100002

**Published:** 2021-11-04

**Authors:** MJ Scarlata, RJ Keeley, SA Carmack, P-J Tsai, JCM Vendruscolo, H Lu, GF Koob, LF Vendruscolo, EA Stein

**Affiliations:** a Neuroimaging Research Branch, National Institute on Drug Abuse, United States of America; b Integrative Neuroscience Research Branch, National Institute on Drug Abuse (NIDA), Intramural Research Program, NIH, Baltimore, MD, United States of America

**Keywords:** Opioid dependence, Opioid addiction, fMRI, Cingulate, Circuit

## Abstract

Opioid use disorder (OUD) is defined as a compulsion to seek and take opioids, loss of control over intake and the development of a negative emotional state when access to opioids is denied. Using functional magnetic resonance imaging (fMRI) data in a rat model of OUD, we demonstrate that the escalation of heroin self-administration (SA) and the increased heroin SA following an injection of an opioid receptor antagonist (naloxone) are associated with changes in distinct brain circuits, centered on the cingulate cortex (Cg). Here, SA escalation score was negatively associated with changes in resting state functional connectivity (rsFC) between the Cg and the dorsal striatum. Conversely, increased heroin SA following naloxone injection, was associated with increased connectivity between the Cg and the extended amygdala and hypothalamus. Naloxone-induced increased SA was also positively associated with changes in the amplitude of low frequency fluctuations within the Cg, a measure of spontaneous neuronal activity. Characterizing the distinct brain circuit and behavior changes associated with different facets of addiction increases our understanding of OUD and may provide insight into addiction prevention and treatment.

## Introduction

In 2019, 2.2% of the US population reported any lifetime use of heroin [1], with ~50,000 people dying from respiratory depression induced by opioid overdose [2]. Heroin is a prodrug for metabolites that bind to the mu-opioid receptor [3]. Heroin use generally begins with drug taking induced intoxication, followed by the rapid development of tolerance and subsequent escalation of intake (reviewed in detail in [4]). Abstinence from heroin results in somatic and affective signs of withdrawal and craving, manifest as dysphoria, physical discomfort, and preoccupation with obtaining more drug. Consequently, opioid use disorder (OUD) is defined as a compulsion to seek and take opioids, loss of control over intake and the development of a negative emotional state when access to opioids is denied. Each stage of OUD is characterized by distinct behaviors and is hypothesized to be driven by changes to discrete brain regions and circuits [5]. For example, the initial binge/intoxication stage is characterized by habit behaviors and involves changes in the basal ganglia [6], whereas the withdrawal/negative affect stage is thought to be characterized by a negative emotional state with changes in, among other regions, the extended amygdala and its interconnections [4,5,7]. Finally, the preoccupation/anticipation stage is often characterized by deficits in executive function, increased drug cravings and ruminations and involves changes in prefrontal cortical circuits [5].

Previous research from our group has identified the extended amygdala and the hypothalamus as responsive to odor cues previously associated with conditioned heroin withdrawal in heroin dependent rats [8]. In that study [8], we used the long (LgA) and short access (ShA) model of heroin self-administration (SA) [9], in which rats are given access to heroin for either 1 h (ShA) or 12 h per SA session (LgA). Rodents exposed to LgA opioid SA exhibit multiple signs of opioid dependence that reflect those that are observed in humans with OUD. These signs include both somatic signs (e.g., wet-dog shakes, diarrhea) and motivational signs (e.g., compulsive-like opioid seeking and taking). However, rodents exposed to ShA opioid SA exhibit significantly less of these signs [8–12], and as such, LgA rats are considered opioid dependent, whereas ShA rats are considered nondependent. In this study, using the behavioral data that we have already published [8], we measured the degree to which a rat increased its heroin intake as compared to its initial intake under ShA or LgA conditions, which was considered a measure of escalation. Next, we measured the SA of heroin following administration of a low dose of naloxone, which competes with opioid agonists at opioid receptors [8]. The amount of heroin taken under this condition was considered a measure of activity of opioid-sensitive neurons. Using this pre-published behavior dataset [8], we now sought to define how intrinsic brain circuits, determined from correlated spontaneous brain activity in the absence of outside stimulation, are altered by heroin dependence and how these circuits relate to these discrete aspects of heroin dependence behavior.

To measure individual differences in intrinsic circuits related to the behavioral characteristics of heroin dependence, rats with a history of heroin SA and naloxone administration under ShA or LgA conditions [8] underwent functional magnetic resonance imaging (fMRI) twenty-four hours after the last SA session. We interrogated functional circuits associated with the two distinct behaviors described above using a ‘seed based’ analysis of resting state functional connectivity (rsFC), a well validated method that presumes that regions with correlated blood-oxygen level dependent (BOLD) signals are functionally coupled [13]. We hypothesized that distinct brain circuits would reflect individual differences in heroin SA escalation trajectory and naloxone-induced increases in SA. In follow up exploratory analyses, we examined alterations in regional coordinated activity using the amplitude of low frequency fluctuations (ALFF) analyses to determine within-region changes in spontaneous brain activity in addition to any functional connectivity changes between regions.

## Methods

### Animals and heroin self-administration

**Fig. 1.**
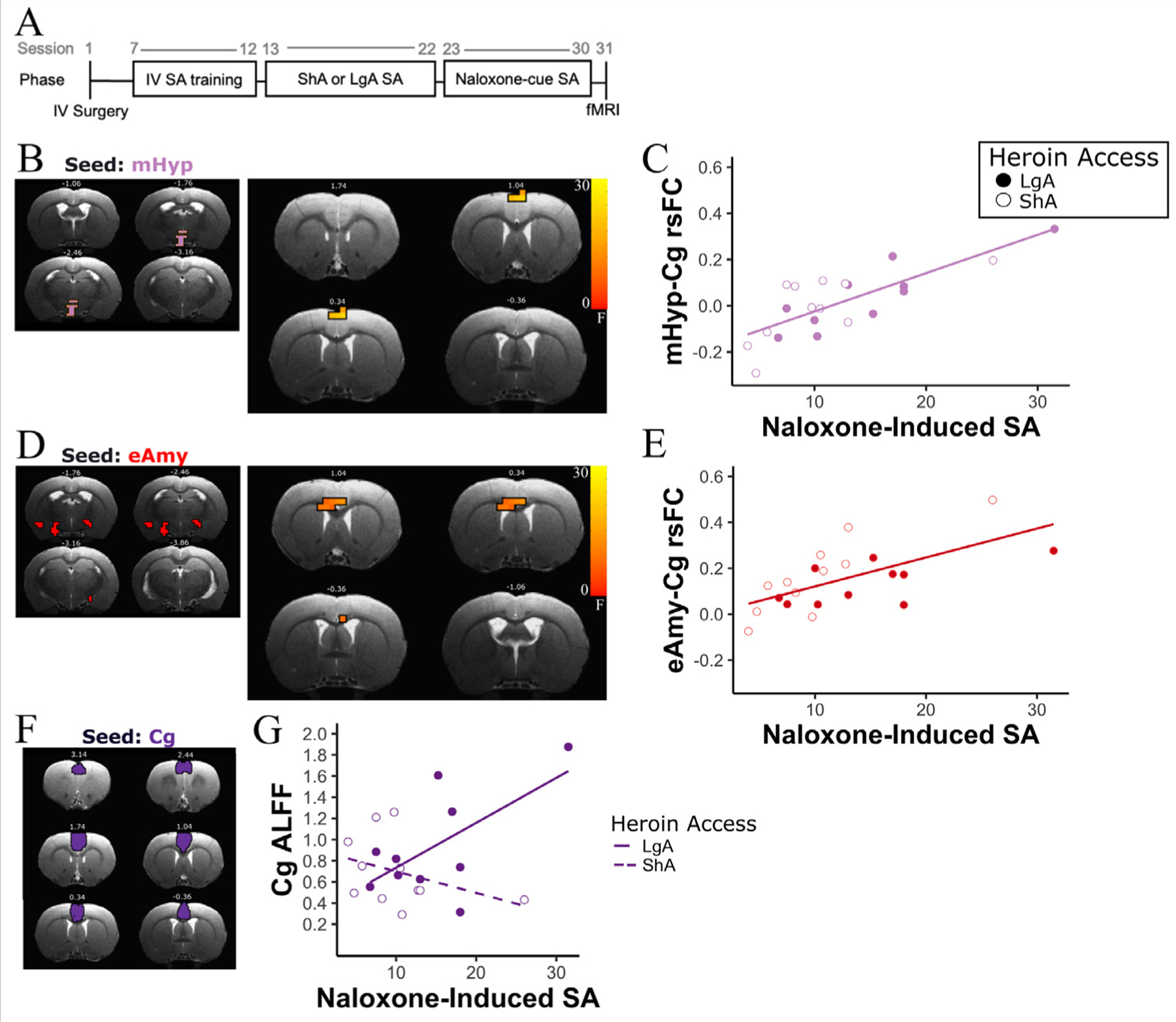
Naloxone-induced increased heroin SA is associated with changes in Cg functional connectivity strength with the eAmy and mHyp and with intrinsic Cg activity A) Experimental timeline. Adult male rats were implanted with a catheter in the right jugular vein (IV Surgery). After a recovery period, they were trained for 6–7 sessions to self-administer heroin (60 *μ*g/kg/infusion) on an FR1 schedule (IV SA training). They were assigned to ShA (1-h SA) or LgA (12-h SA) groups and self-administered heroin over 10 sessions (ShA or LgA SA). The two groups were then injected with either saline or naloxone (120 *μ*g/kg) while being exposed to cues during 8 alternating SA sessions (Naloxone-cue SA). The rats underwent fMRI scanning 24 h after the last SA session. Behavioral data are published in [8]. (B) ANCOVA results (F values) portrayed as a statistical map of the main effect of naloxone-induced self-administered heroin infusions observed on resting state functional connectivity (rsFC) strength between a seed within the hypothalamus (Left panel) and a cluster in the cingulate cortex (Cg) (right panel; p_corrected_ < 0.05; 7 voxels). (C) There was a positive association between number of naloxone-induced self-administered heroin infusions and the rsFC strength between the mid-hypothalamus (mHyp) and the Cg. (D) Statistical map (F values) of the main effect of heroin infusions observed on rsFC between a cluster in the Cg (right; p_corrected_ < 0.05; 7 voxels) and a seed region within the extended amygdala (left). (E) There was a positive association between naloxone-induced self-administered heroin infusions and rsFC between the extended amygdala (eAmy) and the Cg. (F) Anatomically defined Cg ROI [21] used to calculate the amplitude of low frequency fluctuations (ALFF). (G) A significant heroin-access group (LgA versus ShA) x naloxone-induced SA interaction on ALFF within the Cg (F_(1,17)_=6.05, *p* < 0.05) was observed. All statistical maps are superimposed on T2 anatomical coronal images from a representative subject. The numbers above the slices represent the slice’s distance from bregma. Short access SA group (ShA) *n* = 11; Long access SA group (LgA) rats; *n* = 10.

Details about the experimental animals and heroin SA procedures have been previously described by Carmack et al. [8]. The experimental history of the rats is shown in Fig. 1A. Briefly, adult male Long-Evans rats were implanted with an intravenous catheter. After a recovery period, the rats were trained to self-administer heroin (60 *μ*g/kg/infusion) in 1-h sessions under a fixed-ratio 1 (FR1) schedule of reinforcement. Rats were randomly split into ShA (1 h/day) or LgA (12 h/day) SA groups and self-administered heroin over 10 sessions. The two groups were then injected with saline or naloxone (120 *μ*g/kg, s.c.) while being exposed to cues during 8 alternating SA sessions. The rats underwent fMRI scanning 24 h after the last SA session. Behavioral data are published in [8].

### Behavioral scoring

From the behavior reported in [8], two behavioral metrics were created. An escalation score was defined as the average number of heroin infusions during the last three sessions of SA under LgA or ShA conditions only (sessions 20, 21, 22) subtracted by the average number of infusions during the first three sessions of SA under LgA or ShA conditions only (sessions 13, 14, 15; i.e., baseline intake). For the naloxone-induced increases in the SA metric, we used the average number of self-administered heroin infusions following naloxone injections during the first 30 min of each of the naloxone injection sessions.

### Magnetic resonance imaging acquisition

Twenty-four hours following the last odor cue conditioning session, rats underwent fMRI scanning under light anesthesia using a combination of dexmedetomidine (Domitor^®^; Webster Veterinary) and isoflurane (Henry Schein), which has minimal interference on spontaneous brain oscillations [14]. Briefly, anesthesia was initially induced with 2.5% isoflurane and a bolus i.p. injection of dexmedetomidine (0.02 mg/kg). Following placement in an MRI cradle, s.c. dexmedetomidine was continuously infused (0.02 mg/kg/h) in combination with a gradual reduction of isoflurane to 0.75%. Physiological monitoring (HR, temperature and respiration) during fMRI data acquisition was conducted as previously described to maintain neurovascular coupling [8,14–16]; during fMRI acquisition, respiration was maintained at ~65 breaths/min. fMRI experiments were collected within 90 min after the first bolus injection of dexmedetomidine and performed using a Bruker Biospin 9.4T scanner (Bruker Medizintechnik). Following acquisition of high-resolution T2-weighted anatomical images, 3 high spatial resolution resting state functional scans were acquired using a single-shot gradient-echo planar imaging sequence (TR/TE = 1200 ms/13 ms, FOV = 35 × 35mm^2^, matrix size = 64 × 64 (0.547 mm in-plane resolution), slice thickness = 1 mm, 15 slices, 300 time points).

### Data preprocessing

Images were processed using analysis of functional neuro-images (AFNI) software [17,18] and the FMRIB software library (FSL) package [19]. All images were preprocessed using a standard pipeline that included skull stripping, motion correction, registration to a common space, denoising based on independent component analysis (ICA), slice-timing correction, band-pass filtering (0.01 ~ 0.1 Hz) and spatial blurring (full-width half maximum of smooth kernel = 0.6 mm) [15].

### fMRI data analysis

Whole brain, seed-based, rsFC analyses used 3dMVM (AFNI) to compare groups (LgA versus ShA) and either escalation score or naloxone-induced increases in SA score (referred to naloxone-induced SA in figures) as a behavioral covariate. Average respiration rate was included as a covariate to account for differences between individual animal’s respiration rate, shown to be the most critical physiological parameter for fMRI data quality in anesthetized rats [14]. The spatial autocorrelation function (ACF), indicating the degree to which voxels are spatially like one another, referred to as spatial smoothness, was estimated using the 3dFWHMx function in AFNI for each rat’s fMRI image. A smoothing parameter of the averaged ACF across rats was used in combination with the 3dClustSim function in AFNI to estimate the probability of false positive cluster. This was used to estimate the cluster size threshold for a given voxel-wise p value threshold to correct for multiple comparisons. The first-nearest neighbor threshold was used, indicating that voxels were considered part of a cluster if they shared a face. A cluster-level size of 7 voxels set with a corrected p < 0.05 (p_uncorrected_ < 0.01) was considered significant.

The amplitude of low frequency fluctuations (ALFF) [20], measuring regional baseline spontaneous brain activity fluctuations, was computed by voxel-wise Fourier transformation. Then, the square root of the power spectrum across 0.01–0.1 Hz was defined as the ALFF index. Compensating for global variations, normalized ALFF was defined as the output subtracted by mean intensity in the whole brain mask divided by the standard deviation. Following results demonstrating the Cg’s role in dependence behavior (see below), we investigated innate activity differences within the Cg that could be affecting connectivity with brain regions seen in the above rsFC analyses. Therefore, ALFF values were extracted from within the anatomically defined cingulate (Cg) region for each rat. The Cg seed was made up of cingulate area 1 (Cg1) and cingulate area 2 (Cg2) based on a stereotaxic atlas [21]. R studio 1.3.1073 [22] was used to perform an ANCOVA on ALFF within the Cg with heroin access group (LgA versus ShA) as a between-subjects variable and either escalation score or naloxone-induced increases in infusions as a behavioral covariate.

### Statistical analysis

R studio 1.3.1073 [22] was used to perform a Pearson’s correlations examining the relationship between infusions and escalation score.

## Results

### Escalation of drug intake and naloxone-induced increases in SA reflect independent measures

On average, LgA rats had significantly higher escalation scores and self-administered more heroin following a low dose injection of naloxone than ShA rats (data published in [8]). The dose of naloxone was chosen to induce motivational effects of withdrawal (e.g., opioid taking, place-aversion and increased intracranial self-stimulation thresholds) but induce few somatic effects (e.g., shakes and abdominal constrictions) in opioid dependent rats [8,23,24]. There was no significant correlation between escalation score and naloxone-induced increases in SA (*r*=−0.098, *P* = 0.67, CI=−0.51 to 0.35). Given the lack of association between these two behavioral metrics, we considered them independent measures.

### Extended amygdala and hypothalamic connectivity changes with naloxone-induced increases in self-administered infusions

We chose the previously observed [8] mid-hypothalamic (mHyp) region and extended amygdala (eAmy) nuclei, which demonstrated a differential response to conditioned heroin withdrawal cues in LgA rats, as seeds and performed whole-brain connectivity analyses as a function of heroin access (LgA versus ShA) and naloxone-induced increases in heroin self-administered infusions. A one-way ANCOVA using heroin access group (LgA versus ShA) as a between-subjects variable and naloxone-induced SA as a behavioral covariate found a positive association between the circuit strength of the mHyp seed and a cluster within the Cg1 region with naloxone-induced increases in heroin infusions (p_corrected_ < 0.05; Fig. 1B), such that as mHyp-Cg1 connectivity increased, naloxone-induced SA increased (Fig 1C). Similarly, there was a positive association between rsFC strength of a circuit between the eAmy seed and a cluster within the Cg2 with naloxone-induced increases in heroin SA (p_corrected_ < 0.05; Fig 1D), with eAmy-Cg2 connectivity increasing as naloxone-induced SA increased (Fig 1E). No significant connectivity effects were observed due to heroin access group or using escalation score as a factor.

### Intrinsic cingulate activity changes with naloxone-induced increases in self-administrations and heroin access

Given that rsFC between the Cg and both the extended amygdala and hypothalamic seed regions increased with increasing naloxone-induced SA, we next asked whether intrinsic activity within the Cg changed as a function of heroin access and escalation score and/or naloxone-induced increases in heroin SA. We measured the amplitude of low frequency fluctuations (ALFF) [25] within the Cg (combining Cg1 and Cg2 subregions; Fig. 1F) and observed a significant interaction between heroin access and naloxone-induced increases in SA, such that ALFF decreased as naloxone-induced SA increased in the ShA rats, while ALFF increased as naloxone-induced SA increased in LgA rats (Fig. 1G; F_(1,17)_=6.05, *p* < 0.05). Increased ALFF in the Cg of LgA rats suggests increased spontaneous neuronal activity within and between elements in the Cg in dependent rats as naloxone-induced increases in heroin infusions increase. In contrast, there were no main effects of escalation score or heroin exposure group on Cg ALFF.

### Connectivity between the cingulate and the dorsal striatal complex is negatively associated with escalation score

**Fig. 2.**
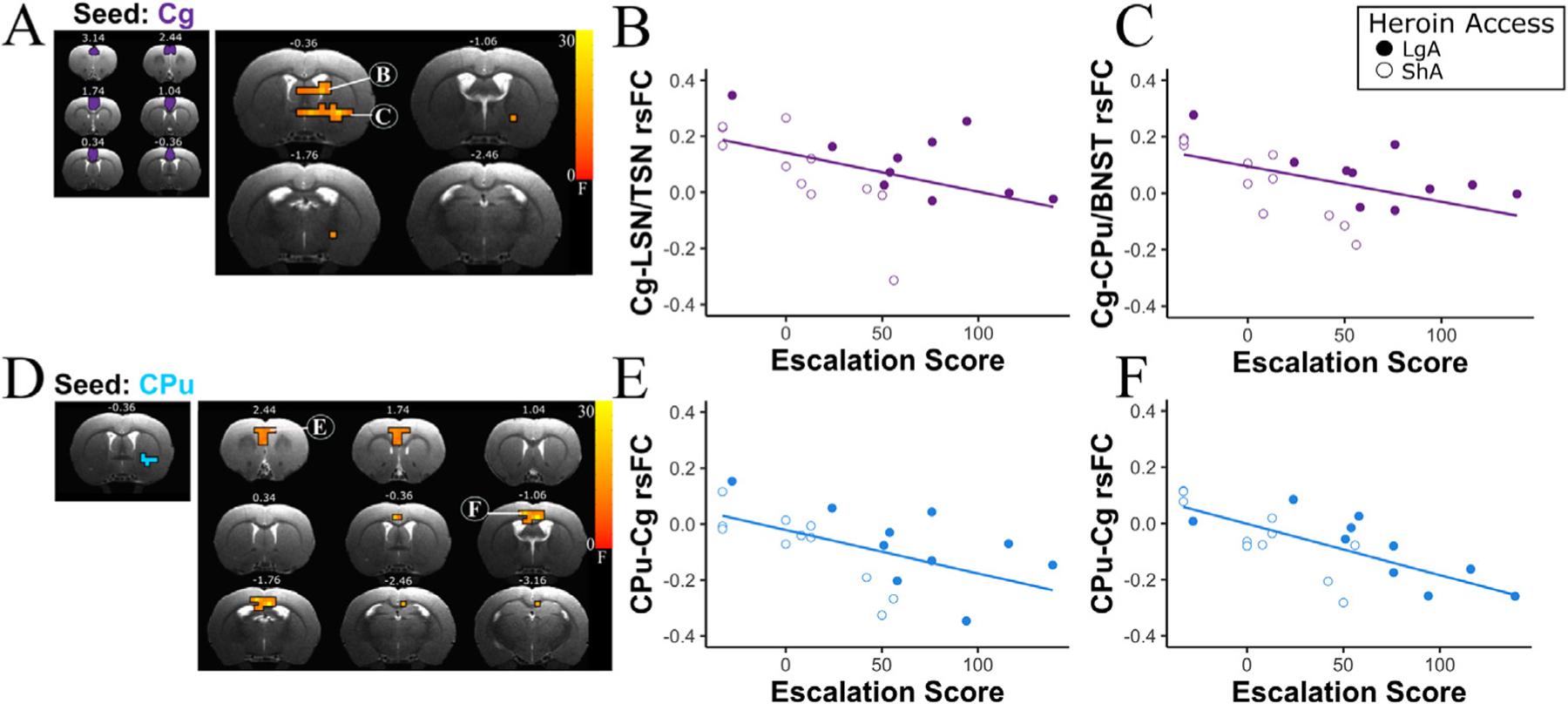
Escalation of heroin self-administration was associated with changes in the functional connectivity strength between the cingulate and striatal regions. (A) ANCOVA results (F values) portrayed as a statistical map of the main effect of self-administration escalation score observed on rsFC between the Cg (left panel) and the lateral septal nucleus/triangular septal nucleus (LSN/TSN) (top; p_corrected_ < 0.05; 8 voxels) and the caudate putamen/bed nucleus of the stria terminalis (CPu/BNST) (bottom; p_corrected_ < 0.001; 15 voxels) (right panel). (B) A negative association was observed between self-administration escalation score and rsFC circuit strength between the Cg and the LSN/TSN. (C) A negative association was observed between self-administration escalation score and rsFC circuit strength between the Cg and the CPu/BNST. (D) ANOVA results (F values) of the main effect of self-administration escalation score observed on rsFC circuit strength between the CPu seed (left panel) and two discrete Cg regions (Cg cluster 1 (E): p_corrected_ < 0.001; 11 voxels; Cg cluster 2 (F): p_corrected_ < 0.001; 15 voxels). (E-F) A negative association was observed between escalation score and rsFC between the CPu and the Cg.

Given that the intrinsic activity of the Cg was altered as a function of ShA and LgA treatment and individual differences in naloxone-induced SA, we next determined whether Cg connectivity with other brain regions was impacted by heroin access and the behavioral metrics of escalation score and/or naloxone-induced increases in heroin SA. Using the Cg (combining Cg1 and Cg2; Fig. 2A) as a seed and performing a one-way ANCOVA with heroin access group (LgA versus ShA) as a between subjects variable and either escalation score or naloxone-induced increased SA as a behavioral covariate, we observed a main effect of escalation score on the rsFC circuit strength between Cg and two clusters: one cluster overlapped with the lateral and triangular septal nuclei, such that there was a negative association between connectivity strength as a function of escalation score (p_corrected_ < 0.05; Fig. 2B), and a second cluster spanned the globus pallidus (GP), caudate putamen (CPu), and the bed nucleus of the stria terminalis (BNST; Fig. 2A), with this circuit’s connectivity strength also negatively associated with escalation score (p_corrected_ < 0.001; Fig. 2C).

Next, given that connectivity between the Cg and these clusters was associated with escalation score but not naloxone-induced increases in SA, we performed a second whole brain rsFC analysis using escalation score as a behavioral correlate with these two identified circuit clusters as seeds. For the CPu/BNST seed, we observed a significant interaction between escalation score and rsFC in a cluster within the Cg (p_corrected_ < 0.001), such that the CPu/BNST-Cg functional circuit strength increased with escalation score. Notably, no changes in rsFC between the LSN/TSN cluster and any other regions as a function of heroin access group (ShA versus LgA) or escalation score were observed (data not shown).

Finally, given the distinct functions and anatomy of the CPu/GP and the BNST in drug dependence (for review, [5]), we conducted an exploratory analysis to determine whether the effects observed between the CPu/BNST-Cg circuit were driven primarily by one of the two identified cluster regions. We anatomically separated the CPu/BNST cluster into the BNST and the dorsal striatum (CPu and GP) seeds based on a stereotaxis atlas [17]. Although no significant circuits were observed using the anatomically separated BNST as a seed, there was a main effect of escalation score using the dorsal striatum as a seed, such that escalation score increased as rsFC between the dorsal striatum and clusters within the Cg decreased (p_corrected_ < 0.001; Fig 2D-F), suggesting that in the originally combined cluster that demonstrated a change in rsFC, the alterations to the connectivity relationship were driven primarily by a dorsal striatal-cingulate circuit. There were no significant effects found as a result of heroin access group (LgA versus ShA) or due to an interaction between heroin access group and escalation score in any of the escalation-related findings.

## Discussion

Using resting state BOLD signal to infer changes in functional connectivity along the trajectory of heroin dependence, we observed that two dependence associated behaviors (heroin escalation and naloxone-induced increases in heroin SA) were associated with distinct cingulate cortex based neural circuits. To do this, we used a priori regions of interest from our previous findings of activity changes in the hypothalamus and extended amygdala that demonstrated differential responses to conditioned opioid withdrawal cues [8]. Independent of heroin access group (i.e., ShA or LgA), we found a strong positive relationship between the number of self-administered infusions following a low, motivation-inducing injection of naloxone and functional circuits to the cingulate cortex from both the a priori amygdala and hypothalamic seeds. It is critical to note that no circuit directionality is implied here and elsewhere when discussing functional connectivity circuits, the circuit is simply denoted from the location of the seed ROI. Given that both withdrawal cue-induced activity based seeds identified circuits with the cingulate, we next interrogated this region, exploring its connectivity with other regions by using the newly identified cingulate as a seed. We found that the escalation in SA was negatively correlated with the strength of two cingulate based circuits: one between Cg and dorsal striatal regions (CPu/GP) and a second between Cg and the lateral and triangular septal nuclei. Moreover, there was an increase in intrinsic activity (ALFF) within the cingulate cortex in the LgA but a decrease in activity in the ShA group as naloxone-induced heroin infusions increased. These distinct, intrinsic cingulate circuit-behavioral associations are reflective of both the multi-faceted nature of dependence and the key role of the cingulate cortex in manifestations of different behavioral aspects of heroin dependence (i.e. escalation score and naloxone-induced increases in SA) in intravenously self-administered heroin-exposed rats using a LgA and ShA model.

Escalation of heroin SA occurs during the period in the addiction cycle where occasional drug use transitions from impulsive to compulsive use [26–28]. Here, Cg-dorsal striatal (CPu/GP) connectivity decreased as individual differences in escalation score increased. There are well known, and reciprocal anatomical connections between the Cg and both ventral and dorsal striatum [29,30]. Both regions are thought to be important components of the Salience Network, a large-scale brain network known to play a role in allocation of cognitive and attentional resources [31,32] and also shown to be modulated during drug dependence [16]. The Cg itself has been implicated in an array of functions, including error awareness [33] and decision making [34], whereas the striatum is well known to convey a reward learning signal [35] and involved in incentive salience and habit learning [for review, see [36].

Connectivity between the cingulate and striatum previously has been strongly implicated in nicotine dependence severity in humans [37,38] and rats [39] such that Cg-striatal connectivity decreased as dependence severity metrics increased. Notably, this circuit was not affected by acute nicotine administration in smokers [38] and the alterations seen in functional connectivity between the Cg and striatum were demonstrated in a rat model to be resultant from chronic nicotine use rather than pre-dispositional [39]. As the directionality of the relationship between Cg-CPu circuit connectivity and escalating addiction-like behavior observed herein concur with that observed in the extant literature [37–39], this circuit has now repeatedly been demonstrated to have an inverse relationship with dependence behaviors across species (FTND score in humans and withdrawal severity score/escalation score in rats) and substances (nicotine and heroin). Moreover, this circuit is unaffected by acute drug challenge with nicotine [40] and only shows altered connectivity associated with dependence behavior following chronic nicotine use [39]. Therefore, the Cg-striatal circuit is worth investigating as a therapeutic target to reduce already escalated drug taking in nicotine, heroin, and potentially other addictive substances

In addition to the Cg-dorsal striatal circuit’s relationship with initial drug use escalation, we observed that increased naloxone-induced heroin SA is associated with increased rsFC between two other Cg-based circuits, one with the eAmy and the other with the mHyp area (Fig 1B-E). Naloxone-induced increases in SA can be used as a metric of opioid receptor sensitivity. The mechanisms of sensitization of MOR signaling are likely multiple. One such mechanism may be heterologous sensitization of adenylyl cyclase (for review, see [41]), a second messenger enzyme that synthesizes cyclic adenosine monophosphate (cAMP). Heterologous sensitization is suggested to occur following persistent activation of G*α*_i/o_-coupled receptors, leading to enhanced cAMP production [41]. In this case, chronic heroin exposure results in prolonged activation of the mu-opioid receptor, due to heroin’s role as a prodrug for metabolites that bind to the mu-opioid receptor [3]. This sensitization has been linked to behaviors that are associated with opioid dependence [42] and may be involved in the alteration in connectivity between the Cg and eAmy and mHyp or vice-versa. Our data implicating the amygdala and hypothalamus in these processes are consistent with the extant literature, as these regions play a significant role in monitoring and processing stress, pain, and negative affective stimuli [43,44]. For example, the hypothalamus is critically involved in the coordination of the endocrine system, moderating the release and inhibition of hormones to maintain homeostasis [45]. In particular, the hypothalamus, in conjunction with the pituitary, is responsible for modulating stress/arousal response. The hypothalamic-pituitary-adrenal (HPA) axis is known to be heavily activated by opioid withdrawal [46] and is hypothesized to contribute to the homeostatic dysregulation that occurs in addiction [47]. Additionally, a pathway between the hypothalamus and the Cg is thought to be involved in the overlap between physical and emotional pain processes that partially compose the reward and withdrawal cycle of addiction [48]. Therefore, opioid-induced changes of Cg-mHyp connectivity may result in dysregulation of stress and pain response in individuals with OUD.

The extended amygdala, a region functionally connected to the hypothalamus [49], is similarly involved in the processing of stress and negative stimuli. During the processing of emotional stimuli, the Cg and limbic areas show increased coupling [43]. Optogenetic inactivation of the Cg projections to the basolateral amygdala inhibit acquisition of observational fear conditioning [44], suggesting this circuit is involved in the acquisition of fear and the resultant stress response when re-exposed to fear-associated stimuli. Notably, in opioid-dependent humans, the amygdala demonstrates volumetric loss and changes in functional connectivity with regions including the anterior insula, nucleus accumbens, and other subcomponents of the amygdala, in comparison to healthy controls [50]. In fact, longer opioid use was associated with greater changes in functional connectivity, suggesting these structural and functional neuronal changes are resultant from long-term opioid use [50]. Taken together, these data suggest that inhibiting one or both circuits may help reduce or bias stress responses following chronic opioid exposure, which might serve to modulate compulsive heroin seeking and propensity to relapse.

Although heroin intake escalation and naloxone-induced increases in SA are associated with distinct neural circuits, these circuits appear to converge on the Cg. Moreover, chronic drug use-induced plasticity not only alters cingulate based circuits (as summarized above) but also modifies intrinsic circuits within the cingulate itself. Here, we assessed spontaneous brain activity fluctuations within the Cg and found that the amplitude of intrinsic low frequency fluctuations (ALFF) increased as naloxone-induced SA increased in LgA rats,whereas ALFF decreased in ShA animals. The Cg is involved in many functions including emotion formation and processing [51], decision-making [34], and interoception [52]. Previous research from our group [16] has shown structural and functional connectivity between the ventral anterior insula and the Cg, which form a hypothesized rat Salience Network. Concurrently, in humans, the Cg has been shown to be involved in error processing in a NoGo/Go response inhibition task [53] and interoceptive feedback [54], two processes that involve the salience network in the human brain.

The Cg, both as a component of the Salience Network and on its own, has been heavily implicated in drug addiction. For example, functional connectivity strength within the Salience Network increases as a function of exposure to a conditioned heroin withdrawal cue in rats with a history of heroin SA [16] and environmental cue-induced activity within the Salience Network of humans was correlated with ratings of craving [55]. Additionally, the Cg cortex of opioid users has reduced concentrations of glutamate/glutamine during a behavioral control task, which occurred in combination with uncoupling of Cg activity and behavioral measures of cognitive control [56], suggesting a dysregulation of the critical role of the Cg in executive function. For instance, Cg activity increases when erroneous responses are given during a response inhibition task and when the correct response is given in the presence of distractions and response competition [33,57]. Together, these findings suggest that the Cg plays a role in predicting errors [58]. Aberrant Cg physiology and activity observed among opioid users may lead to impairments in the ability to detect and/or predict negative consequences of continued drug use [59].

**Fig. 3.**
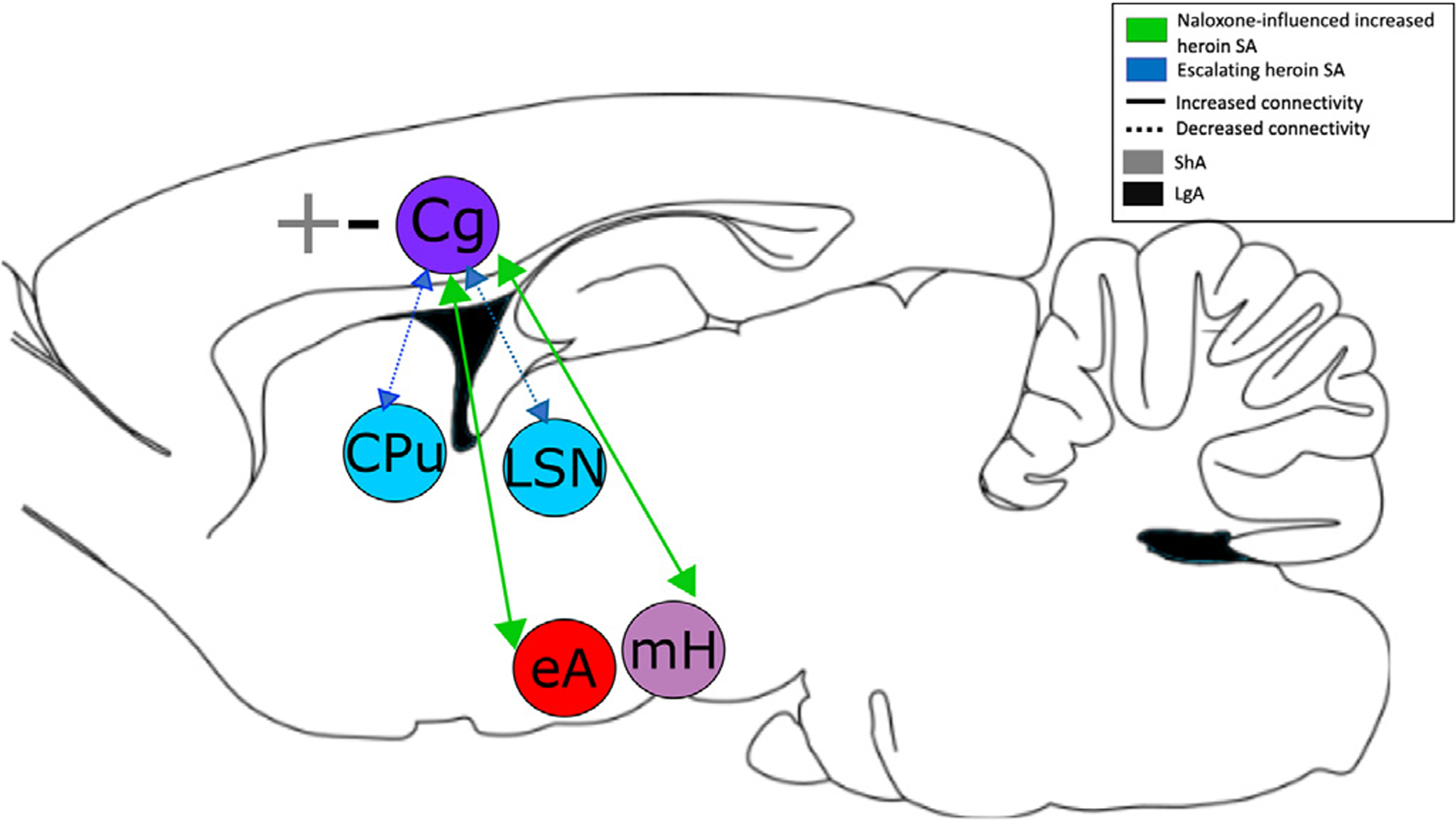
Schematic depiction summarizing the main circuit findings and relationship with addiction behaviors Mid sagittal schematic of the rodent brain highlighting functional connectivity findings as a consequence of heroin SA. Naloxone-induced increased heroin self-administration (green arrows) is associated with greater connectivity between the Cg and extended amygdala (eAmy) and medial hypothalamus (mHyp). Naloxone-induced increased heroin self-administration is also associated with increased amplitude of low frequency fluctuations (ALFF) within the Cg of LgA rats. Escalating heroin use (blue arrows) is associated with decreased connectivity between the Cg and the striatal complex.

Although our data suggest that the distinct circuits, identified here, drive different behavioral facets of opioid dependence, they both converge on the Cg. We propose that as dependence develops, reward-associated Cg-dorsal striatal circuitry contributes to escalating drug taking behaviors. However, following increased opioid receptor sensitivity, Cg-limbic circuits, which are associated with negative emotional learning [7,8], strengthen and are associated with increased heroin use following injection of a low-dose opioid antagonist (Fig. 3). Following heroin escalation, baseline spontaneous activity fluctuations of the Cg increase in lockstep with naloxone-induced increases in SA. This local increase in Cg coherence suggests that drug-induced plasticity is manifest upon local cellular processes within the Cg, which may in turn alter the ability of the Cg to communicate with its efferent and afferent connections. This is consistent with the many functional roles attributed to the Cg and the need to modulate circuit strength to monitor the internal and external environments to adjust behavioral outputs.

Despite the convergence of our findings, multiple study limitations remain. First and foremost, although our sample size was sufficient for the targeted analysis presented here, any network-based analysis would be underpowered and require larger group sizes. Additionally, the behaviors measured represent only a limited sample of addiction-related behaviors and were measured only in male rats. Although the LgA and ShA model of drug use has numerous advantages [23], it does not consider intrinsic differences that may drive drug seeking and taking [60]. Thus, additional dependence models with other parametric behavioral variables that reflect other aspects of this multidimensional disease, and the addition of female subjects should be considered. Similarly, the use of a yoked control in which an animal is given drug in an unpredictable and uncontrollable manner whenever its pair self-administers drug, was not performed. Therefore, we cannot separate out the effects of the compulsive-like extended access SA from other pharmacological effects of the drug when used under conditions such as noncompulsive use of drug for pain control. Also, our model did not directly test for the impacts of genetics, adverse life events, and general lifestyle on addiction behaviors or brain changes. These are all factors known to impact addiction [38,61,62] and thus these cingulate circuits should be additionally examined in diverse populations and environments during opioid exposure. Lastly, our work is correlational and not causational. Additional studies using longitudinal, interventional methods, including optogenetic, chemogenetic, pharmacological and/or non-invasive brain stimulation are needed to determine whether the identified underlying differences in Cg-dorsal striatal, -amygdalar and -hypothalamic circuitry leads to increased risk of dependence or whether drug exposure resulted in alterations within those circuits.

In summary, using a LgA and ShA model of heroin SA, we demonstrate that escalation of SA and naloxone-induced increases in heroin infusions are associated with several neural circuits that center on the Cg, demonstrating its key role in heroin dependence. These results support the hypothesis that pivoting away from the current non-specific, pharmacologic therapeutic approach [63], and towards circuit-based targeted approaches may be useful in treating addiction [64]. Specifically, this work calls for mechanistic investigations into the Cg-dorsal striatal and the Cg-limbic circuits (and potentially their network interactions), and their role in preventing and/or treating dependence to help break the addiction cycle.

## Abbreviations:

LgA: Long access self-administration
ShA: Short access self-administration
Cg: Cingulate
fMRI: Functional magnetic resonance imaging
rsfMRI: Resting-state functional magnetic resonance imaging
SA: Self-administration
eAmy: Extended amygdala
mHyp: Mid-hypothalamus
ACF: Autocorrelation function
ALFF: Amplitude of low frequency fluctuations
OUD: Opioid use disorder
Cg1: Cingulate cortex area 1
Cg2: Cingulate cortex area 2
ROI: Region of interest
ICA: Independent component analysis
